# A Rift Valley fever virus Gn ectodomain-based DNA vaccine induces a partial protection not improved by APC targeting

**DOI:** 10.1038/s41541-018-0052-x

**Published:** 2018-04-20

**Authors:** Tiphany Chrun, Sandra Lacôte, Céline Urien, Luc Jouneau, Céline Barc, Edwige Bouguyon, Vanessa Contreras, Audrey Ferrier-Rembert, Christophe N. Peyrefitte, Nuria Busquets, Enric Vidal, Joan Pujols, Philippe Marianneau, Isabelle Schwartz-Cornil

**Affiliations:** 1 VIM-INRA-Université Paris-Saclay, Domaine de Vilvert, 78350 Jouy-en-Josas, France; 2 ANSES-Laboratoire de Lyon, Unité Virologie, Lyon, France; 3 UE1277-INRA, Plate-Forme d’Infectiologie Expérimentale - PFIE, 37380 Nouzilly, France; 4 CEA-Université Paris Sud-INSERM, U1184, Immunology of Viral Infections and Auto Immune Diseases, IDMIT Department, IBFJ, 92260 Fontenay-aux-Roses, 94270 Kremlin-Bicêtre, France; 5 Institut de Recherche Biomédicale des Armées (IRBA). Unité de Virologie-UMR190, Brétigny sur Orge, France; 6 IRTA, Centre de Recerca en Sanitat Animal (CReSA, IRTA-UAB), Campus de la Universitat Autònoma de Barcelona, 08193 Bellaterra, Spain

## Abstract

Rift Valley fever virus, a phlebovirus endemic in Africa, causes serious diseases in ruminants and humans. Due to the high probability of new outbreaks and spread to other continents where competent vectors are present, vaccine development is an urgent priority as no licensed vaccines are available outside areas of endemicity. In this study, we evaluated in sheep the protective immunity induced by DNA vaccines encoding the extracellular portion of the Gn antigen which was either or not targeted to antigen-presenting cells. The DNA encoding untargeted antigen was the most potent at inducing IgG responses, although not neutralizing, and conferred a significant clinical and virological protection upon infectious challenge, superior to DNA vaccines encoding the targeted antigen. A statistical analysis of the challenge parameters supported that the anti-eGn IgG, rather than the T-cell response, was instrumental in protection. Altogether, this work shows that a DNA vaccine encoding the extracellular portion of the Gn antigen confers substantial—*although incomplete*—protective immunity in sheep, a natural host with high preclinical relevance, and provides some insights into key immune correlates useful for further vaccine improvements against the Rift Valley fever virus.

## Rift Valley fever: DNA vaccines hold promise, but need work

A vaccine made from the genome of Rift Valley fever virus (RVFV) offers partial protection, but pieces of the puzzle are missing, say scientists. French and Spanish researchers, led by the French National Institute for Agricultural Research’s Isabelle Schwartz-Cornil, tested in sheep three slightly-differing vaccine candidates using RVFV genes. Such DNA vaccines are designed to generate proteins which a host’s immune system can use to arm itself against a genuine viral infection. Two of the candidates, designed to target cells that would present the viral proteins to the host’s immune system, provided some benefit to the vaccinated sheep. However, the third untargeted candidate, was the most efficient at protecting sheep, although not completely, and at boosting antibody levels despite not neutralizing the virus. These results provide hope for DNA vaccines against RVFV, and offer direction for future research effort.

## Introduction

The Rift Valley Fever Virus (RVFV) is a zoonotic phlebovirus *(Phenuiviridae*) transmitted by mosquitoes, causing serious diseases in humans and ruminants. In humans, RVFV usually induces a febrile illness, and sometimes hepatitis, hemorrhagic fever, retinitis or encephalitis, leading to death. In ruminants, RVFV is responsible for abortions in pregnant ewes and a high die-off rate in young sheep (see ref. ^1^ for review). RVFV has caused recurrent outbreaks in Sub-Saharan Africa since 1931 and later spread to the Arabian Peninsula, raising awareness of the potentiality for introduction to other continents where a large array of competent insects would embed the virus in the ecosystem with devastating health, social and economic consequences. Attenuated and inactivated RVFV vaccines are commercialized for ruminants in enzootic countries. However, the risk of reversion of attenuated vaccines renders their registration unlikely in Europe and the United States and the need for repeated injections of inactivated vaccines is not compatible with herd management in most countries. The 2017 WHO Blueprint list cites RVFV as one of the 10 priority pathogens for which a low-cost, safe, stable and highly effective vaccine is urgently needed (http://www.who.int/blueprint/priority-diseases/en/).

The M segment of the tripartite negative stranded RVFV RNA genome encodes for a polypeptide generating the Gn and Gc glycoproteins which are involved in viral attachment and fusion, respectively. Neutralizing antibodies (NAbs) are directed to both Gn and Gc.^2^ Gc is located within the glycoprotein shell whereas Gn is exposed on the outside, indicating that Gn is a major target of NAbs.^3^ Importantly, anti-Gn and Gc NAbs can confer protection by passive transfer.^4^ Expression of the Gn ectodomain allowed production of a secreted form (eGn) that, after injection as a protein vaccine in mice, conferred full protection against RVFV,^5^ indicating that eGn is a promising antigen candidate for a subunit vaccine. Furthermore a DNA vaccine encoding eGn was found to be partially protective in mice, despite low levels of NAbs.^6^ Finally, CD8^+^ T-cell responses against the Gn/Gc proteins were identified in the context of protective immune responses,^7^ but their role in protection is unclear.

DNA vaccines present many of the qualities desired for a new RVFV vaccine: they show good stability outside cold chain, they can be prepared for emergency vaccine banks, they are noninfectious and safe, they allow differentiation of infected from vaccinated animals (DIVA), they are easy to construct, their manufacture is feasible at large scale and they have the potential to be cost effective to produce, especially when compared to recombinant proteins.^8,9^ However, whereas DNA vaccines induce high T-cell and Ab responses in rodent models, they usually display low immunogenicity properties in other mammals including ruminants and humans.^10^ Improvement of DNA vaccine immunogenicity has been achieved by enhancing the in vivo delivery with electroporation in skin and muscle,^11^ by adding genetically encoded adjuvants such as granulocyte-macrophage colony-stimulating factor (GM-CSF),^8^ as well as by fusing the encoded vaccine antigen to a single chain fragment variable (scFv), or a chemokine, for targeting a receptor expressed on antigen-presenting cells (APCs).^12–16^ Among APCs, conventional dendritic cells (cDCs) appear as the key cell type involved in APC-targeting efficacy.^17^ In mice cDCs have been classified in two major subsets, the cDC1 and cDC2, with the cDC1 subset excelling in CD8^+^ T and T helper cell type 1 (Th1) CD4^+^ T-cell activation and the cDC2 subset promoting the T helper cell type 2 and 17 (Th2 and Th17) CD4^+^ T-cell differentiation.^18^ Homologous cDC1 and cDC2 subsets have been identified in other species, i.e., in humans, monkey, sheep and pig, and they can be distinguished based on a conserved trans-species phenotype with cDC1 being CADM1^hi^SIRP^lo^ and cDC2 being CADM1^lo^SIRP^hi.^^19,20^ A large number of studies, primarily conducted in mice, revealed that targeting antigens to cDCs using protein or DNA vaccines results in enhancing and even biasing the type of immune responses, depending on both the targeted cDC subset and the nature of the targeted receptor (see refs.^21,22^ for reviews). The most comprehensively studied molecule for APC targeting is DEC205^22^ which is expressed on cDCs in many species including ruminants^23^ and is overexpressed on cDC1s at least in mice. The targeting of different antigens to DEC205 in DNA vaccination of mice^12,13,24,25^ resulted in enhanced Ab, CD4^+^ and CD8^+^ T-cell responses. In addition in a study comparing different targeted receptors using chimeric protein vaccines, CD11c, the general marker of DC, revealed to be a particularly suitable receptor for inducing high humoral responses^26^ and was also efficient at enhancing both Ab and T-cell responses with DNA vaccines in mice.^14^ Despite the spectacular promises from mouse models, the improvement of protective immunity by APC-targeting remains to be documented in large mammals in order to translate this approach to humans and veterinary applications.^27^

Based on this context, we constructed DNA vaccines encoding the RVFV eGn in fusion with a scFv anti-ovine DEC205 and a scFv anti-ovine CD11c and compared them to a DNA vaccine encoding untargeted eGn for their capacity to induce protective immunity in sheep. The vaccines were delivered in the dermis, a tissue densely inhabited with DCs, together with an adjuvant plasmid encoding for ovine GM-CSF.^28^ Surface electroporation (SEP) was used to promote DNA transfection in skin cells and immunogenicity.^29^ Interestingly, we found that the DNA encoding eGn targeted to DEC205 and not to CD11c promoted the interferon-γ (IFNγ) T-cell responses as compared to DNA encoding untargeted eGn, thus indicating that APC targeting can improve T-cell responses outside the mouse species. However, DNA encoding untargeted eGn was the most efficient at inducing anti-eGn immunoglobulin G (IgG) responses, although not neutralizing. This latter plasmid was efficient at inducing significant protection against infectious challenge to a higher extent than the one encoding DEC205- and CD11c-targeted eGn. Furthermore, the anti-eGn IgG levels correlated with protection, suggesting that IgG responses rather than T-cell responses are important in the protective immunity conferred by eGn encoding DNA vaccines.

## Results

### Generation and validation of scFvs for targeting RVFV eGn to ovine DEC205 and CD11c receptors

**Fig. 1:**
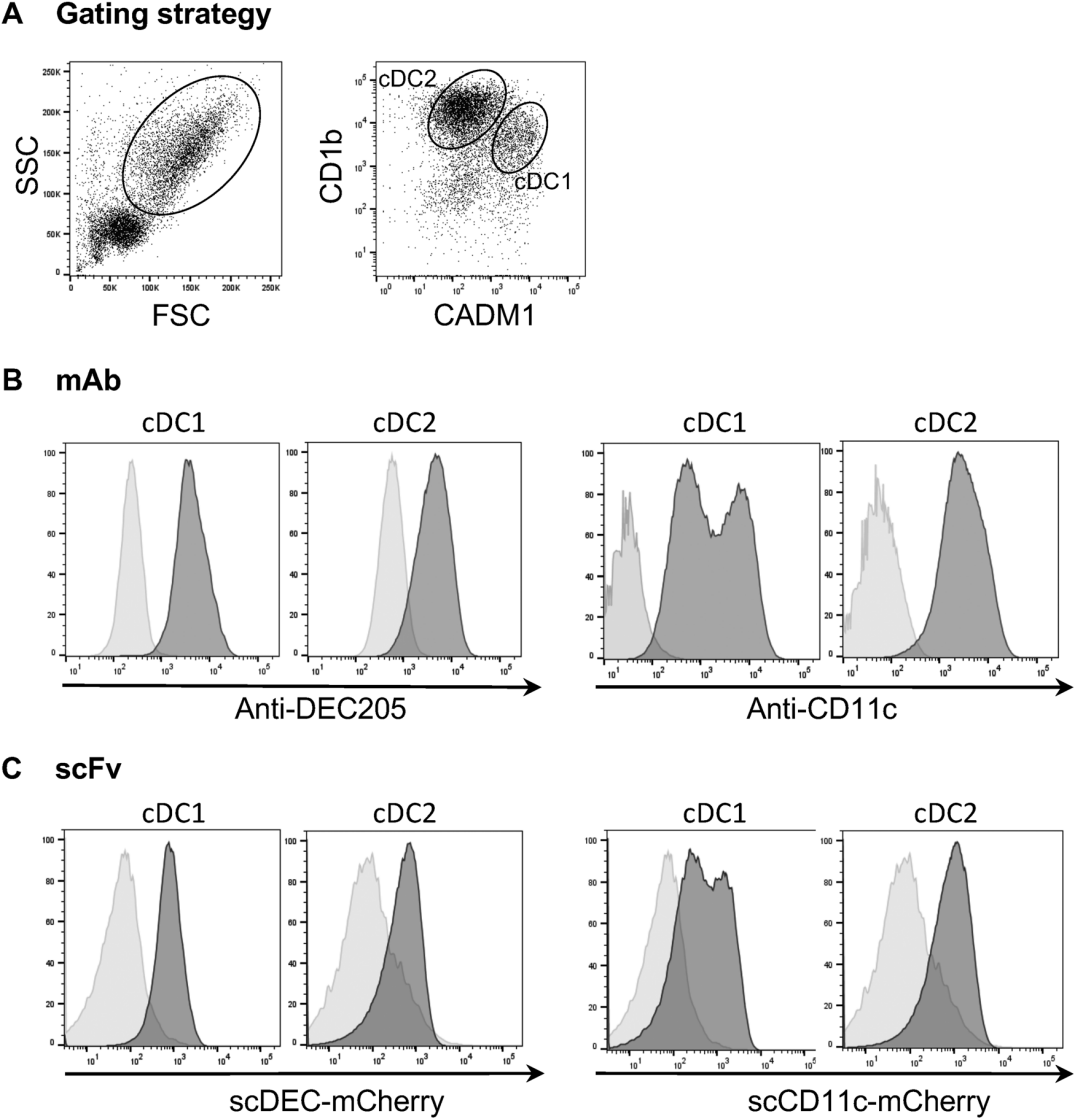
Binding of anti-DEC205 mAb, anti-CD11c mAb, scCD11c-mCherry and scDEC-mCherry on sheep skin lymph DC subsets. **a** Sheep skin lymph low-density cells were gated to select FSC^hi^ cells which were further separated as CD1b^+^ CADM1^hi^ and CD1b^+^ CADM1^lo^ cells, corresponding mainly to cDC1s and cDC2s respectively. **b** The staining of the cDC1 and cDC2 subsets with the parental anti-DEC205 and anti-CD11c mAbs is depicted (dark gray) compared to an isotype control (pale gray). **c** The staining of the cDC1 and cDC2 subsets with scDEC-mCherry and scCD11c-mCherry is depicted (dark gray) compared to a scCtrl-mCherry (pale gray). The shown data are representative of two independent experiments

In order to construct DNA vaccines encoding RVFV eGn targeted to sheep APCs, we derived scFv sequences from two murine hybridomas secreting monoclonal antibodies (mAbs) against ovine DEC205 or CD11c. The scFv sequences were fused to mCherry and the fusion proteins were expressed in HEK293 cells. We evaluated the binding capacities to sheep cDC subsets of scDEC-mCherry and scCD11c-mCherry fusion proteins relatively to a scCtlr-mCherry control, and we compared their staining profiles to the ones of their parental mAb. For that purpose, we collected lymph-draining sheep skin at steady state using the pseudo-afferent lymph cannulation technique^23^ and we isolated low-density cells; these cells are strongly enriched in skin-migrated cDCs which are physiologically relevant cell targets in the context of skin vaccination and which include the cDC1 and cDC2 subtypes, identified as FSC^hi^ CD1b^+^ CADM1^hi^ and FSC^hi^ CD1b^+^ CADM1^lo^ cells respectively (Fig. 1a).^20,23^ A staining with the parental anti-DEC205 mAb shows that cDC1s express slightly more DEC205 than cDC2s, relatively to an isotype control (Fig. 1b), as we previously published.^23^ This finding is in agreement with the slightly higher expression of DEC205 on mouse skin-migrated cDC1s than on cDC2s.^30^ The staining of cDC1s with the parental anti-CD11c mAb appears heterogeneous and presented two peaks, whereas the staining of cDC2 is homogenous. The scDEC-mCherry and scCD11c-mCherry fusions presented a similar staining pattern as the one obtained with the parental mAbs (Fig. 1c). Thus, the anti-DEC205 and CD11c scFvs can be used to create constructs to target RVFV eGn to the corresponding sheep molecules.

### Construction, characterization and expression of anti-DEC205 and anti-CD11c scFvs fused to RVFV eGn

**Fig. 2:**
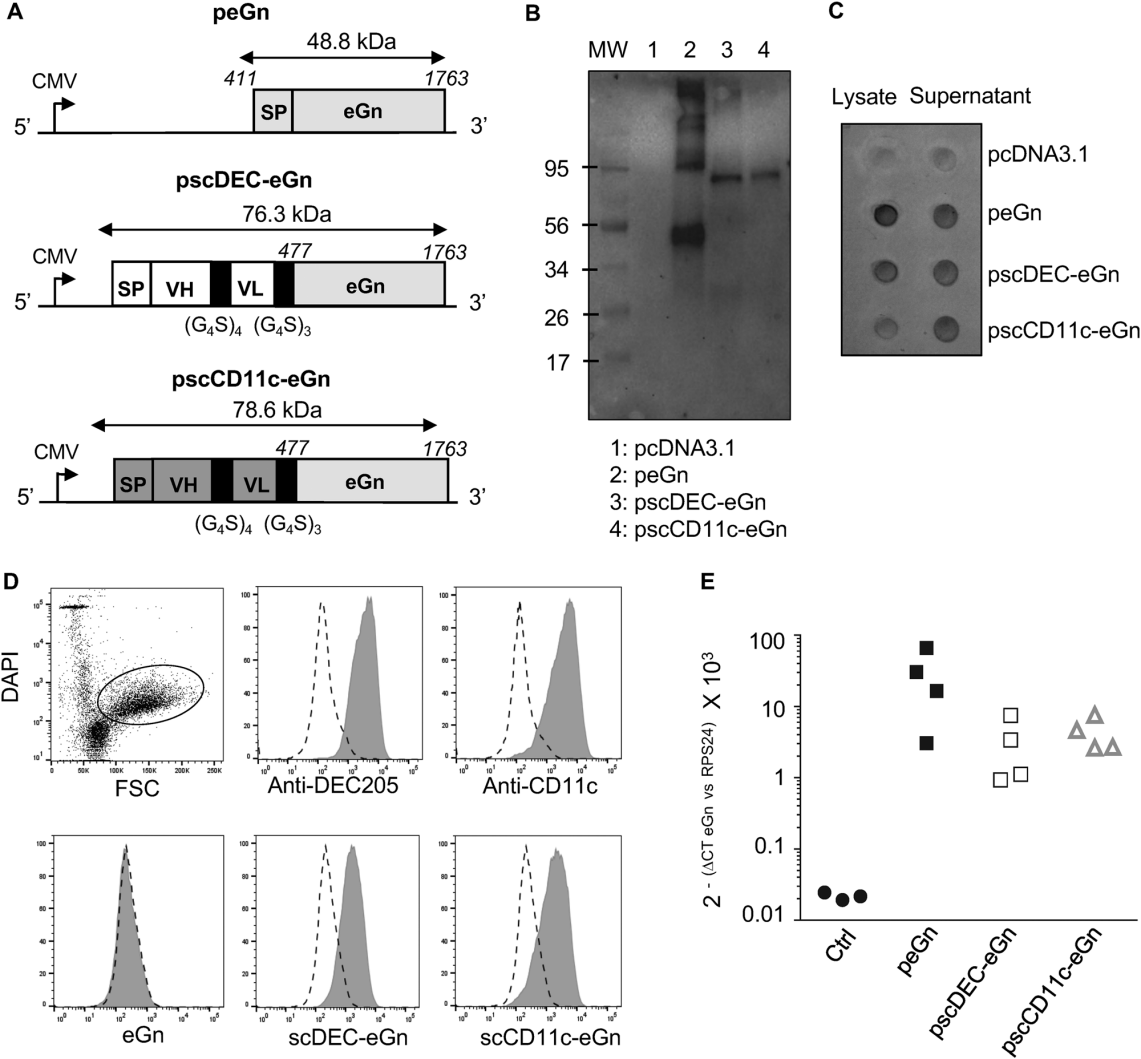
Design and expression of peGn, pscDEC-eGn and pscCD11c-eGn. **a** Schematic representation of peGn, pscDEC-eGn and pscCD11c-eGn. peGn includes the codon-optimized eGn sequence with its intrinsic signal peptide (SP, RVFV M segment from nt 411 to nt 1763) and cloned in the pcDNA4-V5His expression vector. pscDEC-eGn and pscCD11c include the signal peptide (SP) of the VH chain of the parental mAb sequence, the scFv (VH and VL) sequence and the nt 477 to nt 1763 portion of the codon-optimized eGn sequence. The VH and VL sequences are connected together with a (G_4_S)_4_ linker and the scFv and eGn sequences are connected with a (G_4_S)_3_ linker. These chimeric sequences were cloned in a pcDNA3.1 vector. **b, c** HEK293 cells were transfected with pcDNA3.1, peGn, pscDEC-eGn and pscCD11c-eGn. In (**b**), the cell lysates (10 µl) were separated by SDS-PAGE under reducing conditions and eGn was revealed with anti-RVFV HMAF followed by a HRP-GAM IgG. The predicted sizes of the expressed untargeted and chimeric eGn proteins are 48.8 kDa for untargeted eGn, 76.3 kDa for the scDEC-eGn protein and 78.6 kDa for the scCD11c-eGn protein. All samples were gathered from the same experiment and were processed in parallel (1 repeated experiment). In (**c**) the cell lysates (100 µl) and the concentrated cell supernatants (500 µl) gathered from the same experiment were spotted onto the same nitrocellulose membrane and the eGn protein was revealed as in (**b**) (1 repeated experiment). **d** Sheep skin lymph low-density cells were reacted with the parental anti-DEC205, anti-CD11c or isotype control mAbs (top panel) or with the concentrated supernatant from HEK293 cells transfected with peGn, pscDEC-eGn, pscCD11c-eGn and a negative control pcDNA3.1 (bottom panel). The staining of the DAPI^neg^ FSC^hi^ cells, which include dominantly cDCs, is depicted (dark gray) compared to control (dash line). Bound mAbs were revealed with A488-DAM IgG and bound eGn with anti-RVFV HMAF followed by A488-DAM IgG. **e** Relative expression of peGn, pscDEC-eGn and pscCD11c-eGn in sheep skin. peGn, pscDEC-eGn and pscCD11c-eGn (100 µg) were injected intradermally in 100 µl saline in inner front leg sites of two sheep followed by SEP (532 V/cm). After 48 h, RNA was extracted from skin biopsies. eGn mRNA levels were measured using qRT-PCR, normalized with RPS24 ribosomal RNA and expressed as 2^−ΔCT^ values (1 repeated experiment). Absence of residual plasmid in the RNA preparations was controlled using qPCR without reverse transcription

Synthetic genes were designed in order to generate vaccine plasmids encoding untargeted eGn (peGn) and eGn targeted to DEC205 or to CD11c using the validated scFv sequences (pscDEC-eGn and pscCD11c-eGn). As shown in Fig. 2a, peGn includes the eGn sequence (nt 411 to nt 1763 of the RVFV M segment, ZH501 strain) beginning with its natural signal peptide (nt 411 to 477) whereas the plasmids encoding APC-targeted eGn include the VH signal peptide of the original mAb, the scFv sequence and the eGn sequence from nt 477 to nt 1763 which is devoid of transmembrane domains for allowing the fusion protein secretion.

The plasmids were transfected in HEK293 cells and the cell lysates and supernatants were collected for assessing eGn protein expression in vitro. Bands corresponding to untargeted eGn (48.8 kDa) and to scDEC-eGn and scCD11c-eGn protein fusions (76.3 and 78.6 kDa respectively) were detected at their expected predicted size in western blot using an anti-RVFV hyperimmune mouse ascitic fluid (HMAF, Fig. 2b). We could not detect the three proteins in the concentrated supernatants by western blot, possibly due to too limited amounts of eGn that could be detectable by the anti-RVFV HMAF on the nitrocellulose membrane. However, in a dot plot assay where 25 times more volume was loaded, the three supernatants from the eGn-expressing plasmid reacted with the anti-RVFV HMAF (Fig. 2c). Furthermore, when low-density skin lymph cells were incubated with the concentrated supernatants, the binding of scDEC-eGn and scCD11c-eGn could clearly be detected by the anti-RVFV HMAF on FSC^hi^ cells, which mainly correspond to cDCs (Fig. 2d). This experiment indicates that our scFv-based construct can target eGn to sheep skin DCs.

Finally, we assessed whether peGn, pscDEC-eGn and pscCD11c-eGn can be expressed in vivo, and to what relative levels, in the sheep host species. We chose to deliver the plasmids into sheep skin by injection in the dermis followed by SEP, as done previously to successfully immunize mice and pigs with DNA encoding for APC-targeted vaccines.^15,16^ After having determined the optimal plasmid delivery parameters using a firefly luciferase expressing plasmid and escalating electric field values (supplementary material 1), we detected the expression of peGn, pscDEC-eGn and pscCD11c-eGn delivered in sheep skin, using the sensitive real-time reverse transcription-polymerase chain reaction (qRT-PCR) technique, 48 h post administration (Fig. 2e). eGn messenger RNA (mRNA) could be detected in all injected biopsies (from Cq 26 to Cq 32.8, data not shown) and no signal could be detected in the non-transfected skin. After normalization with the RPS24 reference gene, we found that eGn mRNA expression was quite variable for the same plasmid, precluding any statistical analysis. Nevertheless, pscDEC and pscCD11c-eGn tended to be less expressed or were at least not more expressed than peGn in sheep skin (Fig. 2e).

In conclusion, pscDEC-eGn and pscCD11c-eGn express functional fusion proteins capable of targeting eGn to sheep APCs and the mRNA expression of peGn, pscDEC-eGn and pscCD11c-eGn is detectable in pig skin upon electroporation in vivo.

### Immunization with pscDEC-eGn promotes the eGn-specific IFNγ T-cell responses

**Fig. 3:**
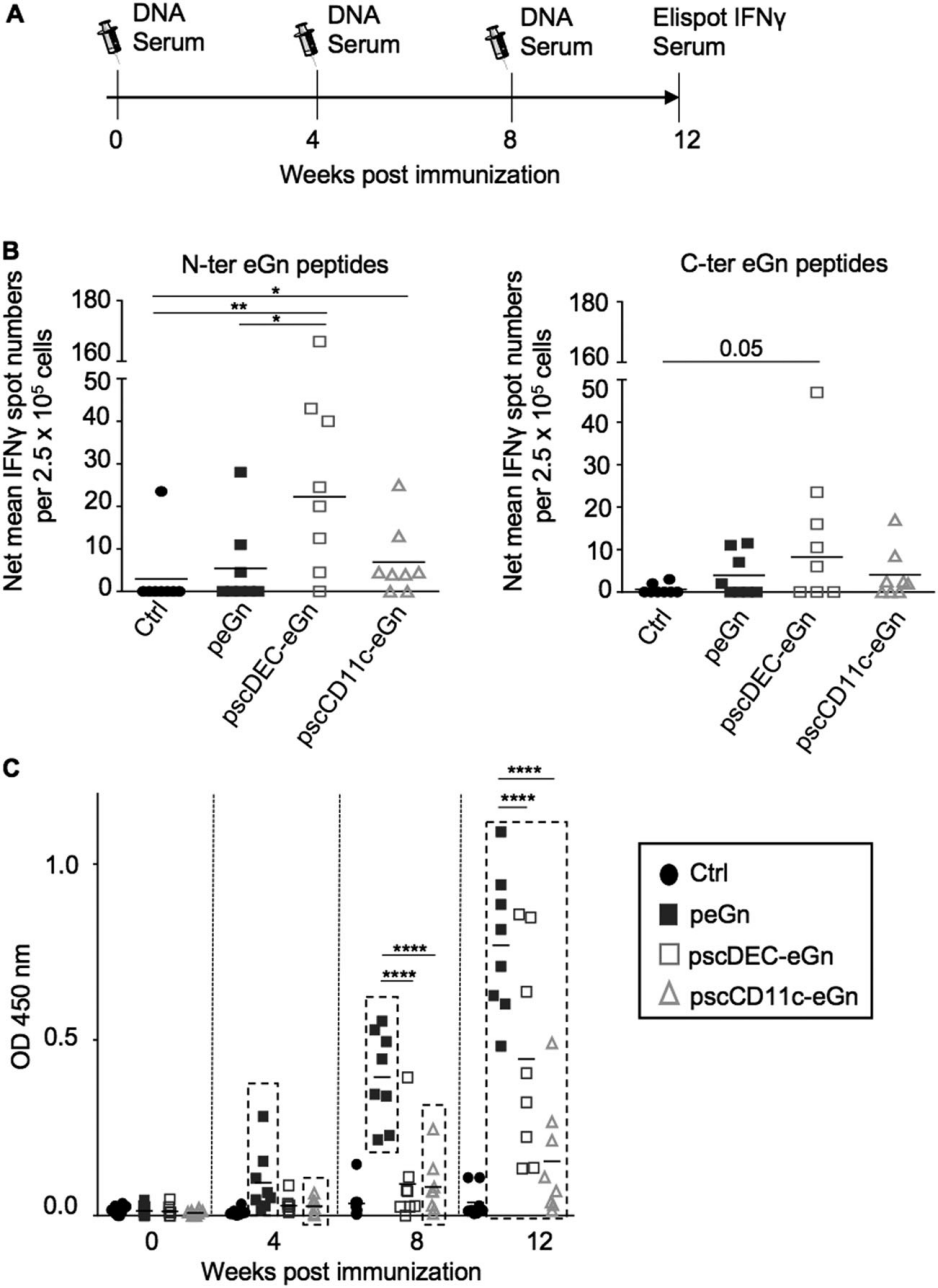
IFNγ T-cell and Ab responses induced by peGn, pscDEC-eGn and pscCD11c-eGn in lambs. **a** Schematic representation of the immunization protocol. The 2-month-old lambs were immunized at weeks 0, 4 and 8 with 400 µg of peGn, pscDEC-eGn, pscCD11c-eGn and 400 µg of pGM-CSF as adjuvant. Control lambs received pGM-CSF only. The plasmids were injected intradermally and the injection was immediately followed by SEP (532 V/cm). Sera were collected and the IFNγ T-cell responses were assessed by ELISPOT at the indicated time points. The syringe drawing is from Servier Medical Art which provides open source illustrations. **b** PBMCs (2.5 × 10^5^) were plated in Multiscreen plates coated with a capture anti-ovine IFNγ mAb (duplicated wells) and re-stimulated for 18 h with overlapping eGn peptides covering the N- and C-terminal parts (5 µg/ml), or with an irrelevant peptide (5 µg/ml). IFNγ spot counts were considered eGn specific when both duplicated well values from cells stimulated with eGn peptides were strictly superior to the duplicated well values from cells stimulated with the irrelevant peptide. The mean spot numbers in stimulated wells minus the ones stimulated with irrelevant peptides are shown (net mean values). Each symbol in the graphs represents an individual animal and the mean is indicated. *P* values between the two groups were determined according to the Mann–Whitney test (*p < 0.05; **p < 0.01). **c** The serum of the lambs immunized with peGn, pscDEC-eGn, pscCD11c-eGn and of control lambs were assayed for the detection of eGn-specific IgG using indirect ELISA. A lysate from HEK293 cells transfected with peGn was used as coating (see Materials and methods section) and the individual sera were tested in a 1:100 dilution. The OD signals at 450 nm from the sera collected at weeks 0, 4, 8 and 12 are shown in 3 C (1 repeated experiment). Each lamb serum is shown as a distinct symbol and the mean OD value is represented as an horizontal bar. *P* values were determined using the two-way ANOVA with Bonferroni’s correction to evaluate the statistical significance of the OD value differences between vaccinated groups (*p < 0.05; **p < 0.01; ****p < 0.0001) and the significance of the differences between the OD values measured at the different weeks versus at day 0 was evaluated for each vaccinated group using paired *t*-test and the groups with statistically significant differences are indicated by a box

The three RVFV DNA vaccines, i.e., peGn, pscDEC-eGn and pscCD11c-eGn, were compared for their immunogenicity in sheep. Each DNA vaccine was combined to pGM-CSF as an adjuvant and was inoculated 3 times intradermally+SEP in 2-month-old Lacaune lambs (Fig. 3a). At week 12 after the prime, peripheral blood mononuclear cells (PBMCs) were re-stimulated in vitro with two pools of overlapping peptides covering the entire eGn sequence (a N-ter and a C-ter peptide pool) in an IFNγ Enzyme-Linked ImmunoSpot (ELISPOT) assay. Cultures with an irrelevant peptide were used as controls (see Materials and methods section). eGn-specific IFNγ responses were observed upon re-stimulation with both the eGn N-ter and C-ter pool peptides in the all-vaccinated groups versus the control sheep groups (Fig. 3b). The most significant difference of response versus the control group was obtained with the cells from the pscDEC-eGn vaccinated sheep (*p* < 0.01 with the N-ter pool and *p* = 0.05 with the C-ter pool). The IFNγ spot number induced by the eGn N-ter pool was significantly higher with the PBMCs from the pscDEC-eGn- than from the peGn vaccinated sheep (Fig. 3b). Although not more expressed than peGn at the RNA level in sheep skin, pscDEC-eGn appears more potent than pscCD11c-eGn and peGn to induce IFNγ T-cell responses.

### Immunization with peGn promotes the anti-eGn IgG responses in sheep sera

Anti-eGn IgGs were measured in indirect enzyme-linked immunosorbent assay (ELISA). The OD450 nm results from 1:100 diluted sera are reported in Fig. 3c. Significant detection of anti-eGn IgG levels was obtained after one injection in the peGn and pscCD11c-eGn-vaccinated group (p < 0.05 in comparisons versus week 0, Fig. 3c). The induction of significant anti-eGn IgG levels was reached for all groups after three inoculations (Fig. 3c). The levels of anti-eGn IgG were higher in the sera from peGn- versus pscCD11c-eGn- and pscDEC-eGn-vaccinated sheep at weeks 8 and 12, and they tended to be higher in the pscDEC-eGn- than in the pscCD11c-eGn-vaccinated sheep at week 12 (Fig. 3c). End-point titers were calculated from serially diluted sera and gave similar differences between groups (Supplementary material 3). The ELISA-positive sera were tested for their capacity to neutralize RVFV in a reduction plaque assay. None of the sera were neutralizing at the difference with positive control sera from infected sheep (data not shown). Overall, there was a graded capacity of our RVFV DNA vaccines for induction of anti-eGn IgG, with peGn being the most potent and pscCD11c-eGn being least potent, and the anti-eGn IgGs were not capable of neutralizing RVFV in vitro.

### peGn and to a lesser extent pscDEC-eGn induce partial protection against infectious RVFV challenge in sheep

**Fig. 4:**
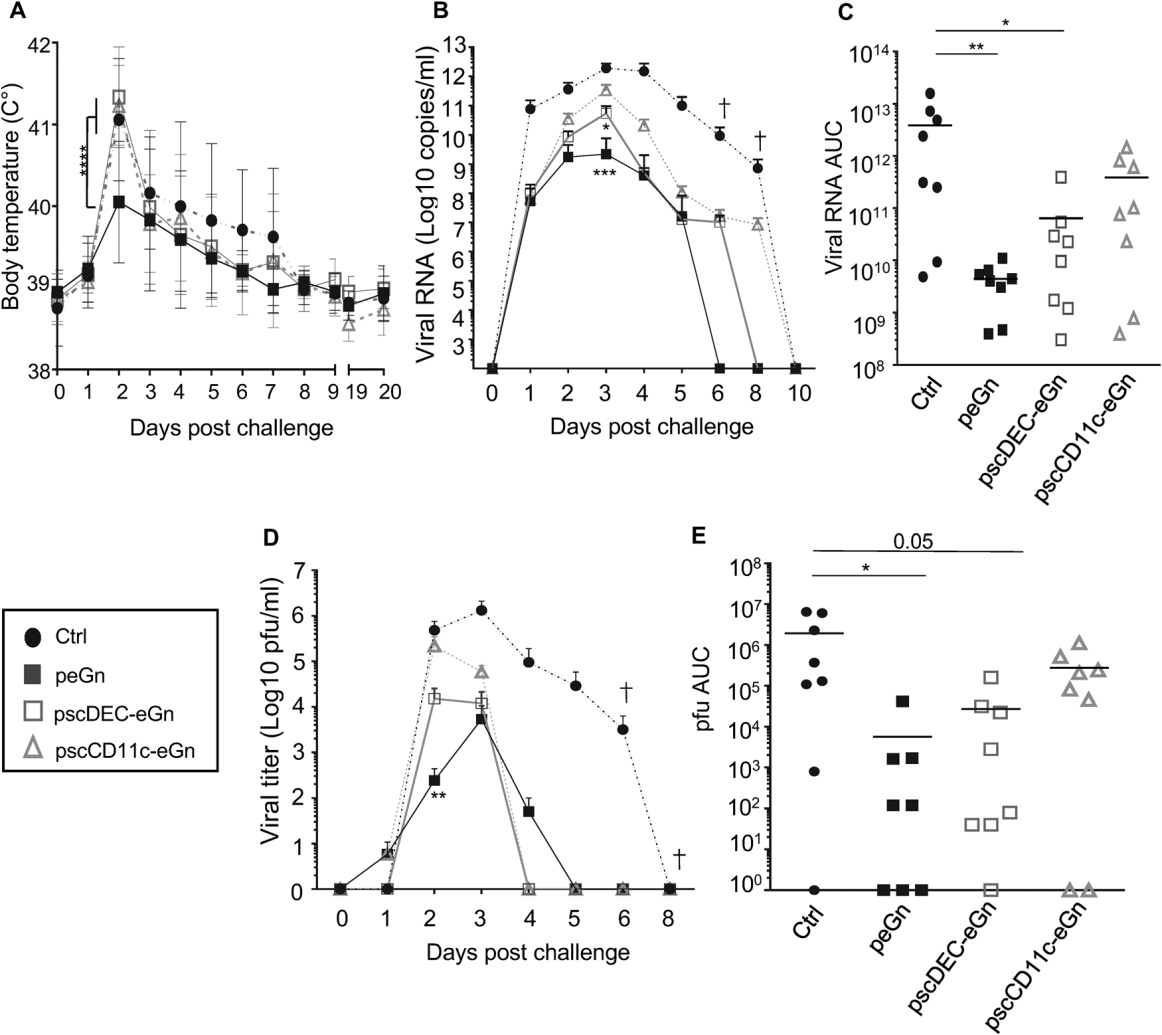
Body temperature and viremia upon RVFV challenge in lambs vaccinated with peGn, pscDEC-eGn and pscCD11c-eGn. The vaccinated and control sheep were challenged with the RVFV 56/74 strain (10^6^ TCID_50_) 3 weeks after the last immunization. **a** The rectal temperature was measured daily. Data are mean ± SD of individual values (*n* = 8 sheep per experimental group). The *p* values were determined according to a two-way ANOVA test with Bonferroni’s correction (****p < 0.0001). **b** The viral RNA copy numbers/ml in serum were quantified by qRT-PCR and the mean values for each group with the top standard error of mean are shown over times. **c** The viral RNA area under the curve (viral RNA AUC) was calculated over 10 days for each animal. **d** The plaque-forming unit load (pfu/ml) were quantified in viral RNA-positive sera and the mean values for each group with the top standard error of mean are shown over times. **e** The pfu area under the curve (pfu AUC) was calculated over 8 days. Each symbol represents an individual animal and the mean is indicated by a horizontal line (*n* = 8 per experimental group). In (**b**–**d**), the *p* values between the vaccinated and control groups were determined with the Mann–Whitney test (*p < 0.05; **p < 0.01; ***p < 0.001). A cross symbol indicates death

Sheep were challenged at week 12 with the RVFV 56/74 strain, a previously demonstrated virulent strain in sheep.^31^ The infected sheep were monitored for clinical symptoms and temperature, and sera were collected regularly for viral RNA and infectious particle detection (see Materials and methods). Two sheep died in the control group at 6 and 8 days post challenge (dpc), and none in the vaccinated groups (Table 1). The post-mortem lesions included severe pulmonary edema, hemorrhages in multiple organs, generalized lymphadenopathy and multifocal liver necrosis. The mean body temperature reached a peak over 41 °C in the control, pscCD11c-eGn and pscDEC-eGn vaccinated groups (41.06, 41.23 and 41.33 °C respectively), whereas the mean body temperature was significantly reduced to 40.05 °C in the peGn vaccinated sheep (*p* < 0.0001) (Fig. 4a). The total clinical score (Table 1), reflecting the severity of the disease (see supplementary material 2 for scoring), was the highest in the control group (92), slightly reduced in the pscCD11c-eGn group (44) and much decreased in the pscDEC-eGn (29) and peGn (22) vaccinated group (Table 1). The main symptoms were nasal mucus discharges, decreased activity, anorexia and conjunctive redness. The viral RNA copies per ml serum were measured on 1, 2, 3, 4, 5, 6, 8 and 10 dpc for each sheep, except for the two dead sheep in which copies were monitored up to 6 dpc (Fig. 4b). The mean integrated value of the viral RNA copies per ml serum (viral RNA area under the curve (AUC), see Materials and methods) reached 3.87 × 10^12^ in the control sheep and was significantly decreased in the pscDEC-eGn and peGn vaccinated sheep (6.39 × 10^10^, *p* < 0.05 and 4.41 × 10^9^, *p* < 0.01 respectively, Fig. 4c). The viral RNA AUC mean value of the pscCD11c-eGn group was slightly but not significantly reduced compared to the one of the control group (3.89 × 10^11^). The peGn vaccinated sheep cleared the viral RNA copies in serum on 6 dpc, the pscDEC-eGn vaccinated sheep on 8 dpc and the pscCD11c-eGn vaccinated and control group on 10 dpc. The particle-forming units (pfu) per ml serum were measured over 8 days (Fig. 4d); the mean integrated values (pfu AUC, see Materials and methods) were calculated and followed the same trend as the viral RNA ones (Fig. 4e). The peGn vaccinated sheep cleared the infectious particles in serum on 5 dpc, the pscDEC-eGn vaccinated sheep and the pscCD11c-eGn vaccinated on 4 dpc and control group on 8 dpc. Finally, the IgG responses directed to eGn, to total RVFV antigens, and to N antigens and the neutralizing Abs (NAbs) were monitored following infection (supplementary material 4). The anti-eGn IgG response was boosted between 5 and 6 dpc in the vaccinated groups, reached the highest levels in the peGn vaccinated sheep (*p* < 0.05), whereas the control sheep developed detectable responses only from 10 dpc, reaching much lower levels than in the vaccinated sheep. The anti-RVFV total antigen IgG followed a similar trend. The titers of the NAbs at 10 dpc reached similar levels in all groups (supplementary material 4), indicating that the DNA vaccines did not induce a clear priming of NAbs, whereas they clearly primed the anti-eGn and anti-RVFV IgG responses which were boosted by the viral challenge. Notably, in agreement with an efficient control of infection by the DNA vaccines, the IgG response against the N immunodominant viral protein, to which all sheep were naive at the time of challenge, was lower in the vaccinated groups than in the control group (*p* < 0.05 at 10 dpc).

**Table 1:** Clinical scores upon infectious challenge in vaccinated lambs

	Individual scores^a^	Total group score^b^	Death per group
Control	21,6,2,13,39,1,1,9	92	2/8
peGn	4,2,4,2,0,8,2,0	22	0/8
pscDEC-eGn	0,11,4,2,7,3,2,0	29	0/8
pscCD11c-eGn	3,8,6,5,5,7,8,1	43	0/8

^a^The individual score was calculated by adding the daily clinical score over 20 days. The daily clinical score was established by monitoring anorexia, eye aspect, activity/movement, lack of rumination, nasal mucus, prostration, respiratory movement, tremor (1 to 3 was attributed depending of severity) and death (15 was arbitrarily attributed)

^b^The total group score was calculated by adding the individual scores of the group

So far, our data show that peGn was efficient at inducing significant levels of protective immunity, reducing by about 3 log10 the RNA viral loads and accelerating viral clearance, in association with a strong decrease in the clinical symptoms severity. pscDEC-eGn, which induced higher T-cell responses and lower anti-eGn IgG responses than eGn, revealed to be less protective than peGn, suggesting that the IFNγ T-cell response is less critical than the Ab response in the protective immunity induced by these vaccines. Finally, pscCD11c-eGn, which was the least efficient at triggering anti-eGn IgG responses, did not induce significant protection.

### The anti-eGn IgG levels at challenge correlate with virological and clinical protection

**Fig. 5:**
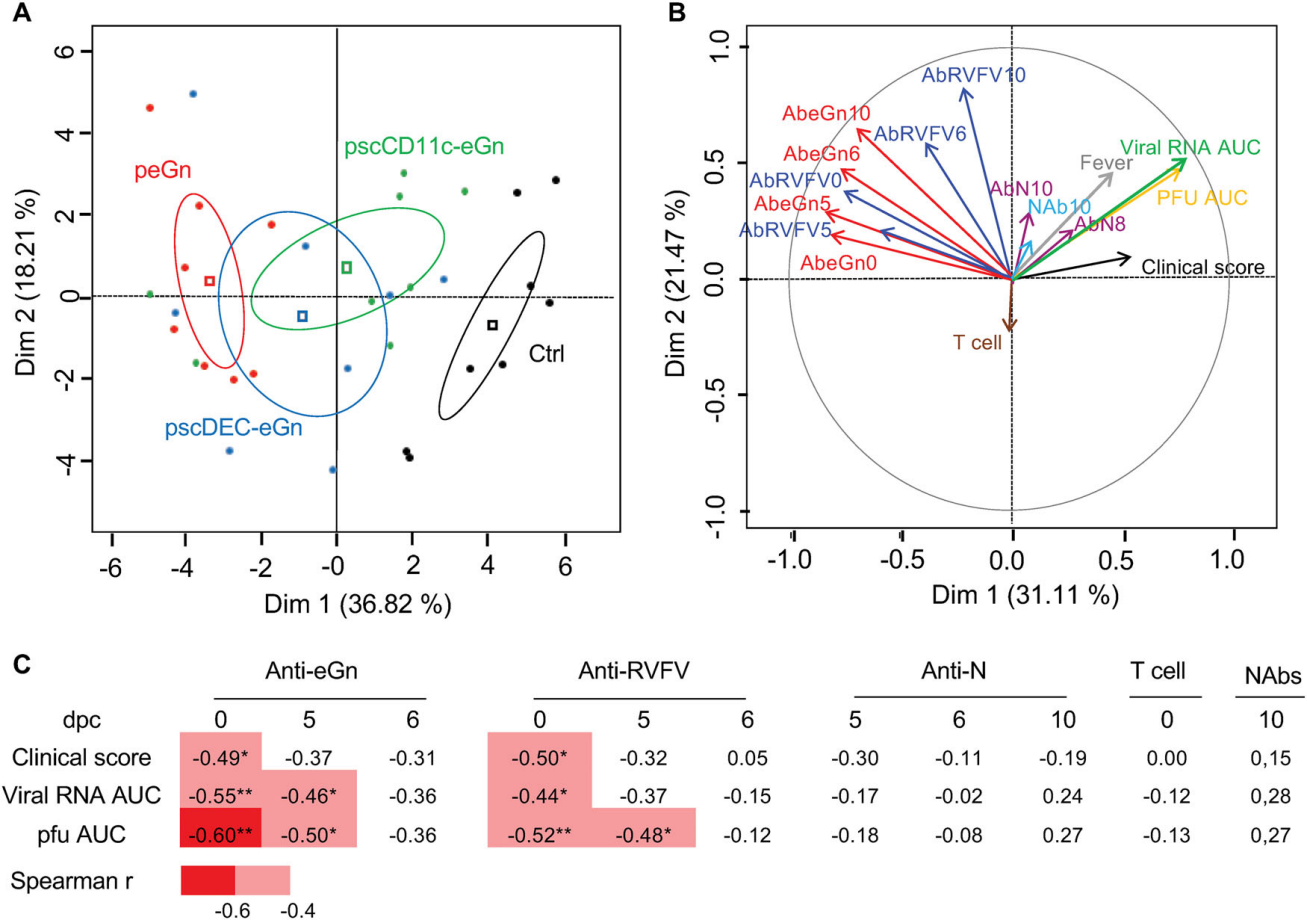
PCA and correlation analysis of the clinical, virological and immune response data of the lambs vaccinated with peGn, pscDEC-eGn and pscCD11c-eGn. **a** A PCA plot of the clinical, virological and immune response data (=PCA variables) of vaccinated and non-vaccinated sheep is depicted, with each sheep represented as a dot in a specific color according to its group assignment: PC1 explains 36.82% of the total variation of the data between sheep and PC2 explained a further 18.21% of the variation. The vaccination regimen is indicated by a distinct color (red for peGn, blue for pscDEC-eGn, green for pscCD11c-eGn). **b** Thirty-one individual input variables were loaded (see Materials and methods) and the most relevant ones are indicated on the figure for clarity: viral RNA AUC, pfu AUC, individual clinical scores, integrated temperature measurements, T-cell responses at week 12, anti-eGn IgG OD signals (AbeGn) at 0, 5, 6 and 10 dpc, anti-N IgG OD signals (AbN) at 8 and 10 dpc, NAb titers (NAb) at 10 dpc and anti-RVFV IgG OD signals (AbRVFV) at 0, 5, 6 and 10 dpc. **c** Correlation analyses between the immune response data versus the clinical and virological data. For each vaccinated lamb, the anti-eGn and anti-RVFV IgG OD signals at 0, 5 and 6 dpc, the NAb titers at 10 dpc and the anti-N IgG OD signals at 5, 6 and 10 dpc were subjected to bilateral Spearman's correlation analysis against the individual clinical scores, the viral RNA AUC and the pfu AUC data. The statistically significant negative correlations are indicated in red (*p < 0.05; **p < 0.01) and the *r* coefficient is indicated

In order to get further insight into the immune parameter(s) associated with protection against RVFV induced by our DNA vaccines, we took advantage of the fact that the three DNA vaccines induced a relatively wide range of values of immune, virological and clinical parameters. These parameter values were loaded in a principal component analysis (PCA) in order to identify the main axes of the data variance as well as the key variables. In a first analysis where we used the data of the control and vaccinated groups, the PC1 axis, which explains 36.82% of the variance, clearly separates the data from the control and the vaccinated group, with the peGn vaccinated group data being the most distant from the control group data (Fig. 5a). In a second analysis using the vaccinated group data, the PCA loading from each parameter shows that the clinical scores, the viral RNA and the pfu cluster together and oppose the anti-eGn and anti-RVFV IgG levels at 0 dpc and less so at 6 and later dpc (Fig. 5b). The T-cell responses, materialized by a short arrow, do not generate any significant pattern (Fig. 5c). Finally, we performed one to one correlation analyses (Spearman's test) and found that the best negative correlation index was obtained between the pfu AUC and the anti-eGn IgG levels at 0 dpc (*r* = −0.6, *p* < 0.01, Fig. 5c and supplementary material 5), and the correlation significance was lost at later time points. A similar negative correlation was obtained between the clinical scores and the anti-eGn IgG levels. No correlation could be found between the T-cell responses and the virological or clinical parameters.

Altogether, the global analysis of the immune, virological and clinical data of sheep vaccinated with peGn, pscDEC-eGn and pscCD11c-eGn indicate that anti-eGn IgG levels at the time of challenge are associated with protection and suggest that these Abs, although not neutralizing in plaque assay, were instrumental in the protective immunity induced by our DNA vaccines.

## Discussion

In this work, we showed that a DNA vaccine encoding untargeted eGn conferred significant protection against a RVFV challenge in sheep. Our finding suggested that the anti-RVFV protective immunity relied on antibodies, although not neutralizing, and not on IFNγ-producing T cells. However, polyfunctional cytokine secretion by T cells and cytotoxicity, which were not assessed here, could also play a role. Importantly, our results indicate that targeting antigens to DEC205 can be used to improve the T-cell response in ruminants when this type of response would be beneficial.

The formalin-inactivated and live-attenuated vaccines have been licensed in African countries where RVFV is endemic (see ref. ^32^ for recent review on RVFV vaccines). However, inactivated vaccines require a booster and annual revaccinations, the live-attenuated Smithburn vaccine is teratogenic and the live-attenuated clone 13, which has a higher safety profile associated to a large deletion in the small segment, can nevertheless induce abortion during the first trimester of gestation.^33^ With the goal to improve safety, next-generation live-attenuated vaccines, such as a reassortant between clone 13 and the MP-12 chemically attenuated strain^34^ or MP-12-derived clone with silent mutations,^35^ have been developed. However, there is resistance of many countries to authorize live-attenuated vaccines, due to the risk of reversion to virulence. Therefore, other vaccine candidates were generated and demonstrated promising results in sheep and include subunit vaccines,^36,37^ virus-like particles,^36^ virus replicon particle vaccines,^34^ virus-vectored vaccines^36,38,39^ and DNA.^40^ In contrast to our results, a DNA vaccine encoding for the glycoprotein precursor Nsm/Gc/Gn did not induce T- or B-cell response in sheep, using 3 injections of 400 µg DNA in lipofectin as a delivery method.^40^ Several of these novel candidates were compared to a commercial vaccine, either to an inactivated vaccine^36^ or to clone 13.^34^ In the first study, the inactivated vaccine decreased by 4 log10 the peak of viral RNA levels in serum and was less efficient than purified eGn in oil-in-water adjuvant or than a viral replicon encoding for Gn/Gc.^36^ In the second study, clone 13 and the viral replicon induced full protection without detectable viral RNA in serum.^34^ However, it should be pointed out that the mean RNA copies per ml serum at the peak of infection of control sheep was close to 8 log10 copies in the first study^36^ and to 10 log10 copies in the second one,^34^ whereas it was above 12 log10 copies in our study, suggesting that our challenge conditions were more severe. We can speculate that a milder challenge would have improved the reduction of viral RNA load induced by our DNA vaccine (about 3 log10 here). Nevertheless, caution should be taken to compare these studies which were performed with different sheep breeds and different viral strains. Therefore, efforts should be made to better standardize across labs challenge experiments that should include a commercial vaccine as a reference to identify promising vaccines.

Higher levels of anti-eGn IgG were reached with peGn than with pscDEC-eGn and pscCD11c-eGn and correlated to protection. The higher Ab quantity could result from a higher expression of peGn in vivo, a hypothesis which is supported by the tendency for higher mRNA expression from peGn in skin; however, an analysis of protein expression from a large number of transfected skin biopsies would be necessary to further validate this hypothesis. In addition, untargeted eGn may be available to B cells for a longer time than the DC-targeted eGn fusions which are likely to be rapidly secreted and readily internalized by the targeted receptor, a feature which has been proposed to reduce Ab responses while promoting T-cell responses.^15^

Besides quantity, the quality of the antibodies generated by our different DNA vaccines may also play a role in protection. In that respect, the fact that the anti-eGn antibodies induced by all three DNA vaccines were not neutralizing in vitro is puzzling. In addition, our DNA vaccines did not appear to prime for neutralizing antibody production since the DNA vaccinated sheep and control sheep showed similar levels at 10 dpc. In contrast, soluble eGn produced by drosophila cells and delivered in a oil-in-water adjuvant could induce neutralizing antibodies in sheep^36^ but in line with our finding, a DNA vaccine encoding untargeted eGn administered to mice using gene gun in skin conferred significant protection with weak induction of neutralizing antibodies.^6^ The eGn protein, related to its mode of expression by skin cells, may not be in an optimal conformation to induce neutralizing antibodies. Alternatively, the capacity of antibodies to neutralize the infectivity of RVFV may be different in the gold standard assay using VeroE6 cells and in the in vivo conditions, due to the likely difference of viral receptor engagement, as proposed in the case of flaviviruses.^41^ The anti-eGn antibodies induced by DNA vaccines may display anti-RVFV properties such as complement-dependent inhibition or Ab-dependent cellular cytotoxicity (ADCC) or phagocytosis. The ADCC efficacy depends on the Ab isotype and on the nature of the recognized epitope on the viral protein, as shown in the case of influenza.^42^ Untargeted eGn and eGn fused with scFv may differ in their respective capacities to generate such anti-viral antibodies. Assessment of the anti-RVFV Ab functions will require the development of suitable in vitro tests which would help to potentially identify better correlates of protection than anti-eGn Ab levels.

pscDEC-eGn did promote the IFNγ T-cell responses in sheep, although it tended to be less expressed than peGn in sheep skin. In the context of DNA vaccination, DEC205 targeting appeared to have a positive effect on T-cell responses in several instances such as in mice^12,13^ and even cattle,^43^ but in other cases DEC205 targeting rather decreased the IFNγ-producing T-cell response with regulatory T-cell induction.^44,45^ Here, the targeting of eGn to CD11c did not promote T-cell responses, although pscCD11c-eGn was at least as expressed as pscDEC-eGn. This is in contrast with results in mice where CD11c targeting strongly enhanced the IFNγ T-cell responses.^14^ Therefore, the outcome of DEC205 and CD11c targeting of antigens expressed by DNA plasmids is inconsistent and difficult to predict for T-cell as well as for B-cell responses,^12,44^ in a given species and throughout species, and could depend on several parameters. Firstly, DC activation by the DNA delivery method is a prerequisite to induce strong T-cell responses, as the targeting of steady-state DC induce regulatory T cells and tolerance;^46^ therefore, the DNA delivery method, the combination with adjuvants and the duration of plasmid expression could all affect the efficacy of DC targeting. In the present work, we combined electroporation which has been proposed to have a strong adjuvant effect through inflammatory cytokine release and APC mobilization in skin,^29^ with a plasmid encoding ovine GM-CSF, a largely used cytokine adjuvant in DNA vaccination including in sheep.^8,28^ Secondly, the nature of the targeted vaccine antigen, by its content in self-mimicking epitopes expanding regulatory T-cell responses, may affect effector T-cell priming.^47^ Thirdly, the same targeted orthologous receptor may have different subcellular routing pathways with variable kinetics in different species, impacting on the efficacy of major histocompatibility complex (MHC) class I or class II antigen presentation,^17,48^ and even in the same species, Ab targeted to different epitopes on the same molecule were shown to induce different responses.^49^ Finally, the distribution of expression of the targeted receptor among cell types and mononuclear phagocyte subsets in different species may affect the immune response, given the functional specialization of the different subsets. Indeed, in humans and ruminants, DEC205 expression on leukocyte populations is much broader than in mice and is found on B, T and NK lymphocytes.^50,51^ In mice, CD11c is expressed primarily on DCs and also on some macrophages and natural killer (NK) cells, whereas in sheep CD11c is widely expressed on macrophages and B cells.^52^

To the best of our knowledge, our study is the first to demonstrate that a plasmid encoding for eGn induces a significant protection in sheep, indicating that an effective vaccine against RVFV could be produced based on DNA. However, our strategy needs to be further optimized. Indeed, for application in the field and especially in Africa, the minimum number of administrations and the plasmid dose to achieve protection should be established with downscaling experiments, and the mode of delivery should be adapted to mass vaccination, such as with a high pressure needle-free delivery system which has been shown to be as efficient as electroporation in pigs^16^ or possibly with new portable electroporation-based systems.^53^ The level of protection that we achieved is incomplete and complete immunity is desirable to protect the highly sensitive fetuses; however, a strong decrease in viral load would also decrease the risk of transmission to mosquito vectors. The immunity triggered by peGn might be further improved by adding other RVFV antigens such as by adding Gc (or eGc) and/or N proteins. While the T-cell response against eGn did not seem to impact protection, T-cell responses against other RVFV antigens may be of value. Better vectors driving higher antigen expression would be also desirable: indeed, differences in expression between vectors, as suggested by Fig. 2e, may have masked a positive effect of the targeting. Finally, as our results support the importance of anti-eGn IgG in protection, strategies to further improve this type of response should be privileged. In that respect, eGn could be targeted to receptors such as CLEC9A which is a particularly suitable targets for Ab response induction.^54,55^ CLEC9A is selectively expressed on cDC1s in different species including in sheep,^23^ and its targeting results in the prolonged antigen presentation favoring follicular helper T-cell development, strong germinal center formation, leading to high Ab responses of strong affinity.^54^ However, despite the existence of a CLEC9A ortholog in sheep and high expression of mRNA by cDC1s,^23^ we were unable to generate any anti-CLEC9A mAb reacting with sheep cDC1s. Alternatively, XCR1 targeting, another receptor exclusively expressed by cDC1s across species, could also be used to successfully induce high anti-eGn IgG responses in sheep, as recently showed by us in pigs for promoting anti-influenza M2e IgG responses.^56^

In conclusion, we showed that DNA vaccination is a very promising control strategy against RVFV in sheep. In addition, by using CD11c and DEC205 targeting which was detrimental to protection, we found that Ab responses against eGn, and not IFNγ T-cell responses, are significant correlates of protection in sheep. This outcome of DC targeting related to its capacity to modulate immune responses could be extended to other pathogens for identifying immune correlates of protection in application species where mechanisms are difficult to investigate. In the case of RVFV, this DC-targeting strategy guides us towards the development of future vaccines optimized for induction of high levels of anti-eGn IgG whose anti-viral functions remain to be deciphered.

## Materials and methods

### Abs and hydridomas

The hybridomas producing the anti-ovine CD11c (mouse IgG1, OM1) and the anti-ruminant DEC205 (mouse IgG2b, CC98) mAbs were developed by INRA and the Pirbright Institute (former Institute for Animal Health (Compton, UK)) respectively. The mAbs used in this study are the anti-CD1b (mouse IgG2a, Th97, WSU (USA)), the anti-CADM1 (chicken IgY, 3E1, CliniScience, #CM004-3), the anti-bovine IFNγ (CC330, Bio-Rad, #MCA2112) and the biotinylated (biot)-anti-bovine IFNγ (CC302, Bio-Rad, catalog #MCA1783). The conjugated antibodies are the Alexa 647-conjugated (A647)-donkey anti-chicken IgY (Jackson ImmunoResearch, #703-606-155) and the A488-donkey anti-mouse IgG (DAM) (Jackson ImmunoResearch, #715-546-150), the A488-goat anti-mouse (GAM) IgG2a (Fisher Scientific, #10114282), the phycoerythrin (PE)-GAM IgG1 (Fisher Scientific, #10246642) and PE-GAM IgG2b (Fisher Scientific, #10012802), and the horseradish peroxidase (HRP)-rabbit anti-mouse IgG (Sigma-Aldrich, #A9044) and HRP-mouse anti-sheep IgG (Sigma-Aldrich, #A9452). The anti-RVFV HMAF production was approved by the French Ministère de la Recherche et de l’Enseignement Supérieur under the number 02518.2 and was conducted at ANSES, Lyon, France. Balb/c mice were inoculated intraperitoneally with inactivated RVFV-infected mouse brain suspension in Freund’s complete adjuvant on days 0 and 1, in Freund’s incomplete adjuvant on days 7 and 14, and with a virulent suspension in Freund’s incomplete adjuvant on days 21 and 28. On day 25, the mice were inoculated intraperitoneally with TG180 sarcoma cells. After 1 week, ascitic fluid was harvested.

### Cell lines

Human embryonic kidney cells (HEK293, ATCC® CRL-1573) and African green monkey kidney cells (VeroE6, provided from Pierre Russo, Anses, Sophia-Antipolis Laboratory, Ruminant Pathology Unit, 06902, France) were maintained in culture with Dulbecco’s modified Eagle's medium (DMEM, Life Technologies), supplemented with 5% decomplemented fetal bovine serum (FBS) and antibiotic solution at 37 °C. Cell lines were checked to be free of mycoplasma contamination using a mycoplasma detection Kit (MycoAlert™, Lonza, Switzerland).

### RVFV strains

The virulent strain RVFV 56/74, provided by Alejandro Brun (CISA-INIA), originally isolated from an infected cow,^31^ was amplified on C6/36 insect cells and titrated on VeroE6 cells according to the Reed–Muench method. The attenuated RVFV virus MP-12 strain,^57^ provided by Michèle Bouloy (Paris Pasteur Institute), was amplified and titrated on VeroE6 cells.

### Construction and production of plasmids

Genes encoding for the variable region of the murine heavy (VH) and light (VL) chains of the anti-ruminant DEC205 and the anti-ovine CD11c mAb were amplified by PCR and subcloned in the pSK vector for sequencing (Synogene). The sequences were deposited on Bankit (https://www.ncbi.nlm.nih.gov) under the ID 2070859 (release on 1 September 2018). For each mAb, the original VH signal peptide was fused to the VH and the VL fragments joined by a sequence encoding for a flexible linker (G_4_S)_4_ to generate the scFv sequences. A sequence encoding for the ectodomain of the Gn glycoprotein (eGn) of the ZH501 strain was codon-optimized for expression in eukaryotic cells (Genecust, Luxembourg) and was fused in frame to the carboxy terminus of the scFv sequence with a (G_4_S)_3_ linker. The eGn sequence spans from nucleotide (nt) 477 to nt 1763 (DQ380202.1) of the RVFV M segment, and is devoid of transmembrane domains. The fusion constructs encompassing the VH signal peptide, scFv and eGn were cloned into a pcDNA3.1 expression vector (InvitroGen) and the resulting plasmids are designated as pscDEC-eGn and pscCD11c-eGn. In addition, fusion constructs with mCherry replacing eGn sequences were built as well as a construct including a control scFv, provided by Synogene (pscDEC-mCherry, pscCD11c-mCherry and pscCtrl-mCherry). A plasmid encoding for untargeted eGn was generated (peGn). peGn includes the nt 411 to nt 1763 codon-optimized sequence of the M segment with the natural signal peptide of Gn^58^ cloned into a pcDNA4-V5His expression vector (InvitroGen) (Genecust). The plasmid encoding for ovine GM-CSF (pGM-CSF) has been previously described.^28^ A firefly luciferase expression plasmid (pLuc) was kindly provided by Stéphane Biacchesi (INRA, France). The sequence encoding the RVFV N nucleoprotein was cloned into the expression vector pMT/BiP/SNAP-His. Plasmid productions for immunization were prepared using Endofree® Plasmid Giga Kits (Macherey-Nagel) and were stored at −20 °C until use.

### Plasmid transfection in vitro

HEK293 cells (5 × 10^6^ cells) were transiently transfected with JetPRIME® (Polyplus, USA) according to the manufacturer’s protocol. Briefly, 10 µg of peGn, pscDEC-eGn, pscCD11c-eGn or pcDNA3.1 (negative control) were mixed with 20 µl of polymer reagent and added to the cells. The supernatants and cells were harvested at 72 h post transfection.

### Cell lysate and supernatant preparation

Cell pellets of transfected HEK293 cells or MP-12-infected VeroE6 cells were lysed in borate lysis buffer (1% Triton X-100, 50 mM borate, 150 mM NaCl, pH 9)+protease inhibitors at a 1:9 volume ratio (pellet/buffer), the lysates were sonicated, aliquoted and kept frozen. The transfected cell supernatants were concentrated 20 to 40 times with Amicon® Ultra centrifugal filter 30 K (Millipore) and a protease inhibitor cocktail was added.

### Western blots and dot blots

Cell lysates were analyzed by western blot following a 12% sodium dodecyl sulfate–polyacrylamide gel electrophoresis (SDS-PAGE) under reducing conditions and cell supernatants were analyzed by dot blot under nonreducing conditions. Blots were incubated overnight at 4 °C with anti-RVFV HMAF in a 1:1000 dilution in phosphate-buffered saline (PBS)+10% fat-free milk (w/v) followed by HRP-rabbit anti-mouse IgG Ab. Immunoreactive bands were visualized with ECL substrate (SuperSignal™ West Femto, Thermofisher) and dots were visualized with 3,3′-diaminobenzidine substrate (Sigma-Aldrich). Image acquisitions were done with Chemidoc imaging system (Bio-Rad). The blot experiment was performed with respect to the ethical guidelines and the technical protocols conducted at ANSES laboratory in Lyon, France.

### Lymph duct cannulation and low-density lymph cell collection

The prescapular lymph duct of Prealpes sheep was cannulated to collect pseudo-afferent lymph cells draining from the skin, as previously described.^59^ The cannulated sheep were housed at the Unité Commune d’Expérimentation Animale in Jouy-en-Josas, France. This protocol was done under compliance with national legislation on animal experimentation and was approved by the Committee on the Ethics of Animal Experiments of the INRA research center in Jouy-en-Josas (COMETHEA) under the number 11–019.

### Assessment of plasmid transfection efficacy in sheep skin

Plasmid administration in sheep skin was performed on Prealpes sheep (2 to 12 months old) under general anesthesia (2 mg/kg xylazine and 10 mg/kg ketamine, intramuscular route). Immediately after intradermal injection of DNA in the left and right inner front leg zones, SEP was performed on the site of injection using the CUY 21 EDIT system (BEX, Tokyo, Japan). Disk electrodes (10 mm) were loaded with conductive gel (Alcyon, France) and 6 electric pulses were applied during 10 ms with 90 ms interval. In order to evaluate the V/cm parameter leading to optimal transfection in sheep skin, a pLuc reporter vector (100 µg) was injected intradermally and the injected skin areas were subjected to SEP with escalating pulses from 0 to 1331 V/cm. After 2 days, skin punch biopsies were harvested and lysed with 150 µl of lysis reagent (Luciferase Assay System E1500, Promega). Bioluminescence was measured using the In Vivo Imaging System (IVIS-200, Xenogen, UK) after adding 100 µl luciferine substrate. The optimal V/cm was found to be 532 V/cm and was used in all subsequent experiments (supplementary material 1). This experiment was approved by the COMETHEA ethic committee under the number 201504211534406 (APAFIS#520) in accordance with national guidelines on animal use and performed at the Unité Commune d’Expérimentation Animale UCEA-INRA in Bressonvilliers, France. In order to compare the expression of the different vaccine plasmids in sheep skin in vivo, plasmids (100 µg) were injected intradermally and subjected to SEP (532 V/cm). After 2 days, sheep were killed, 4 skin biopsies were collected from each injection site and frozen in liquid nitrogen. This experiment was approved by the French ethical committee CEEA Val de Loire, received the authorization number 04150.02 in accordance with national guidelines on animal use and was conducted at the Plate-Forme d’Infectiologie Expérimentale PFIE-INRA, Nouzilly, France.

### Immunizations and RVFV challenge

The experiment was approved by the Spanish ethical committee Department Medi Ambient i Habitatge de la Generalitat de Catalunya CE-8820 and was conducted at IRTA-CReSA, Barcelona, Spain, under compliance to Spanish biosafety and ethic rules. Lambs (2 months old) were equally distributed in both sexes and were housed in the IRTA-CReSA experimental farm in Lerida, Spain, under A-BSL1. Lambs were randomly divided into 4 groups of 8 lambs and the vaccination was performed 3 times at 4 week intervals (0, 4, 8) under general anesthesia (2 mg/kg zoletil, intramuscular route); 8 animals per group is classically used in vaccine studies. Then, 400 µg of peGn, pscDEC-eGn or pscCD11c-eGn were injected intradermally together with 400 µg of plasmid adjuvant (pGM-CSF), followed by SEP as described above. A non-vaccinated control group (PBS plus pGM-CSF) was included. Sera were collected at weeks 0, 4, 8 and 12 post immunization and PBMCs were harvested at week 12 to analyze the T-cell response. The immunized sheep were moved to a A-BSL3 to perform the challenge at day 91 after the first immunization with 10^6^ TCID_50_ of the RVFV 56/74 strain in 2 ml volume by subcutaneous route. After challenge, the clinical signs were daily monitored in a blinded manner. The daily clinical score per sheep was established by monitoring anorexia, eye aspect, gait, lack of rumination, mucosa aspect, prostration, respiratory movement, tremor (1 to 3 depending of severity) and death (15) (supplementary material 2). An individual clinical score per sheep was obtained by adding the daily values over 20 days. The final global score per group was the addition of each individual score. Serum was collected at 0, 1, 2, 3, 4, 5, 6, 8, 10, 12, 14, 16, 18, 20 dpc. At the end of the study, the animals were killed by an overdose of sodium pentothal.

### Low-density lymph cells and PBMC isolation

Low-density lymph cells were enriched over an optiprep gradient as previously described by our group,^59^ frozen in FCS+10% DMSO using a Nalgene Mr. Frosty Cryo 1 °C Freezing Container and kept in liquid nitrogen. PBMCs were isolated in the A-BSL3 IRTA-CReSA’s facility using 8 ml Vacutainer® CPT™ (BD Bioscience) by centrifugation at 1800 × *g* for 35 min at 25 °C. Tubes were shipped overnight at 4 °C to INRA, Jouy-en-Josas, France. Upon arrival, PBMCs were washed with PBS+1.3 mM citrate and re-suspended in X-vivo medium (Ozyme, France) supplemented with 50% FBS for overnight recovery at 4 °C.

### Staining of lymph dendritic cell and flow cytometry analysis

Low-density lymph cells were thawed and incubated on ice with PBS supplemented with 4% horse serum for 30 min. To analyze DEC205 and CD11c expression on DC subsets, lymph cells were labeled with a mix of primary mAbs (2 µg/ml) for 30 min, being either anti-CD1b+anti-CADM1+anti-DEC205 mAbs, or anti-CD1b+anti-CADM1+anti-CD11c mAbs. Appropriate isotype control mAbs were used. Cells were washed and finally incubated with a mix of secondary Abs (1:200) being respectively A488-GAM IgG2a+A647-anti-chicken IgY+PE-GAM IgG2b or A488-GAM IgG2a+A647-anti-chicken IgY+PE-GAM IgG1. To analyze the binding of scDEC-mCherry and scCD11c-mCherry, the fusion proteins (50 µg/ml)+anti-CD1b+anti-CADM1 mAbs were incubated with lymph cells on ice for 30 min. A scCtrl-mCherry provided by Synogene was used as control. Cells were washed and incubated with PE-GAM IgG1+A488-GAM IgG2a. To analyze the binding of scDEC-eGn and scCD11c-eGn on low-density lymph cells, the concentrated supernatants of HEK293 transfected cells with peGn, pscDEC-eGn, pscCD11c-eGn and pcDNA3.1 (negative control) were incubated with cells for 2 h at 4 °C. Cells were then incubated with anti-RVFV HMAF (1:1000) and finally with A488-DAM IgG (1:200). Dead cells were excluded by 4',6-diamidino-2-phenylindole (DAPI) staining. Flow cytometry acquisitions were done with a LSR Fortessa flow cytometer (Becton Dickinson) and results were analyzed using FlowJo 10.0.6 software.

### Evaluation of eGn-specific T-cell responses by ELISPOT

IFNγ-secreting T cells were detected using polyvinylidene difluoride membrane-bottomed 96-well plates (Multiscreen®_HTS_, Millipore) coated with 15 µg/ml anti-bovine IFNγ (capture mAb) in PBS. PBMCs (2.5 × 10^5^) were added to wells and re-stimulated with 5 µg/ml overlapping peptides (20 mers, offset 8, Mimotopes, Australia) covering the N-terminal (AEDPHL […] LLPDSF) and C-terminal (VCFEHK […] NYQCHT) amino-acid sequence of the eGn protein in X-vivo medium (Ozyme, France) supplemented with 2% FBS and antibiotic solution at 37 °C for 18 h. The overlapping peptides were divided in 2 pools initially diluted in H_2_O/acetonitril (5 mg/ml) and distributed in 5 µl per well to have 1 µg of each peptide per well and avoid any toxicity of the H_2_O/acetonitril diluent. In addition, cells incubated with 5 µl of H_2_O/acetonitril peptide diluent (1:1 volume) and with an irrelevant peptide derived from the HIV polymerase (IKDFHVYFRESRDALWKGPG) were used as negative controls. Cells cultured with ConA (25 µg/ml) were used as positive controls. Each condition was tested in duplicated wells. IFNγ-secreting T cells were revealed using biot-anti-bovine IFNγ mAb at 0.5 µg/ml followed by alkaline phosphatase-conjugated streptavidin (Mabtech, Sweden) and BCIP/NBT reagent (Life Technologies). Plates were automatically counted using the iSPOT AID ELISPOT reader. IFNγ spot counts were considered eGn specific when both duplicated well counts from cells stimulated with eGn peptides were strictly superior to the duplicated well counts from cells stimulated with irrelevant peptide. The net mean of the IFNγ spot counts were obtained by subtracting the mean counts from wells stimulated with irrelevant peptides out of the mean counts from wells stimulated with eGn peptides.

### Evaluation of anti-eGn, anti-N and anti-RVFV responses by ELISA

High-binding 96-well plates Nunc Maxisorp^TM^ (ThermoFisher) were coated overnight at 4 °C either with 100 µl lysates from MP-12-infected VeroE6 cells or from HEK293 cells transfected with peGn or with SNAP-tag purified N protein, which was produced using a Drosophila expression system and kindly provided by Philippe Despres (Paris Pasteur Institute, France). The same lysates and protein batch was used in all experiments. The same positive and negative sheep sera were used to validate each plate. Sera were diluted 1:100 in PBS+2% fat-free milk (w/v) and incubated on the coated plates at 37 °C for 1 h. The plates were subsequently incubated with HRP-mouse anti-sheep IgG in a 1:4000 dilution at 37 °C for 1 h. HRP enzymatic activity was revealed using TMB (3,3′,5,5′-tetramethylbenzidine) substrate. Optical density was measured at 450 nm using a TECAN microplate reader. Corrected OD values were obtained by subtracting the signal of wells coated with lysates from uninfected VeroE6 cells, from HEK293 cell transfected with pcDNA3.1, or coated with SNAP-tag. To calculate anti-eGn IgG end-point titers, 1:100 diluted sera were serially diluted 1:1 and the ELISA was processed as described above. End-point titers were calculated with a regression analysis plotting dilution versus *A*_450_ as the highest dilution giving twice the absorbance of the mean of negative control wells.

### RNA extraction from serum and transfected skin samples

Total RNA was extracted from post-infection sera with QIAMP kit (Qiagen) according to the manufacturer’s instructions and eluted in 60 µl RNase-free water using QIACube (Qiagen). Total RNA was extracted from skin biopsies collected from the inoculation sites (about 250 mg per site) using 10 ml TRIzol LS reagent (Thermo Fisher Scientific) and homogenization with Ultra-Turrax T8. The first RNA extract was purified using a RNeasy MinElute cleanup column. The 1 µg purified RNA was treated twice with 5 µl of RNAse-free DNAse (30 Kunitz units, Qiagen) in order to remove any residual DNA plasmid from the injection and subjected to a final purification on column.

### Gn RNA quantification by qRT-PCR

For quantification of viral RNA copies per ml sera, a one-step qRT-PCR was performed using the SuperScript III Platinum One-Step qRT-PCR kit (Invitrogen) as previously described.^60^ Primers were designed to amplify a nt 1164 to nt 1258 Gn sequence of the M segment: forward primer 5′- AAA GGA ACA ATG GAC TCT GGT CA–3′, reverse primer 5′- CAC TTC TTA CTA CCA TGT CCT CCA AT-3′. A fluorescent probe was designed that hybridizes to the specific PCR product: FAM 5′- AAA GCT TTG ATA TCT CTC AGT GCC CCA A-3′ TAMRA. A standard corresponding to nt 1135 to nt 1286 of the M segment was synthetized using the Riboprobe® In Vitro Transcription Systems (Promega). The qRT-PCR was done on 3 µl of the serum elution and the program was 45 °C for 30 min, 95 °C for 5 min, linked to 45 amplification cycles of 95 °C for 5 s and 57 °C for 35 s, and cooling at 37 °C for 30 s. TaqMan run of experimental samples contained at least two replicates, a known positive control (RVFV ZH501 strain), a negative control (an other Phlebovirus RNA) and nuclease-free water. The number of viral RNA copies/ml in each serum sample was determined using the Gn RNA standard calibration curve. For each animal, the AUC of the viral RNA copies per ml over 10 days (viral RNA AUC) was calculated. For the detection of Gn mRNA in transfected skin biopsies, a two-step qRT-PCR was performed. The reverse transcription was done using the TaqMan® Reverse Transcription Reagents (Applied Biosystems). In order to check that the transfected skin RNA did not include detectable residual plasmids, reverse-transcribed (3 ng estimated cDNA) and non-reverse transcribed RNA (3 ng RNA) were subjected to PCR reaction with the iTaq UniverSYBR Green (Bio-Rad) for amplifying a Gn fragment with forward primer 5′ – AGT GCG ATG GGC AGT TGT C–3′ (M segment, codon-optimized sequence) and reverse primer 5′ – TTC TTG AAC ACG GCA AAT GG–3′. The PCR cycling conditions were 95 °C for 10 min, linked to 40 cycles of 95 °C for 15 s and 60 °C for 1 min. No residual plasmid could be detected in any of the RNA samples. The relative expression of Gn mRNA in transfected skin samples was normalized using the qRT-PCR signals obtained with the ovine *ribosomal protein 24* (*RSP24*) gene which is a stably expressed reference gene in sheep cells.^61^

### Virus titration by plaque assay

The sheep sera were serially diluted in DMEM medium and inoculated onto VeroE6 cells in 12-well plates for a plaque assay over 5 days. The pfu were revealed by 1% (w/v) violet crystal and counted. For each animal, the integrated pfu load corresponding to the AUC of the number of pfu per ml serum over 8 days was calculated (pfu AUC).

### Plaque reduction assay

Neutralizing antibody was measured using plaque reduction and neutralization test. Sera were diluted 1:10 to 1:10^4^ in DMEM and incubated with 100 pfu of MP-12 virus at 37 °C for 1 h to enable neutralization to occur. Next, the virus and the serum mix (500 μl) were added to VeroE6 cell monolayers for a plaque assay. The neutralizing antibody titers were established as the last dilution which inhibited 50% of the foci number per well compared to virus-only control titration.

### Statistical and correlation analysis

Data were analyzed with the GraphPad Prism 6.0 software. To compare the T-cell responses, the anti-eGn IgG titers, the NAb titers at day 10, the viral RNA at day 3, the pfu at day 2, the viral RNA AUC, the pfu AUC values and the luciferase signals between the two groups, we used the unpaired non-parametric Mann–Whitney test, as the data did not pass the normality test (Kolmogorov–Smirnov test). The paired parametric *t*-test was used to compare the antibody responses at different time points versus at day 0 within the same group. A two-way analysis of variance (ANOVA) test followed by Bonferroni’s correction was used to compare the OD values of the Ab responses and the body temperature between immunized groups each day after infection, as the data distribution passed normality test. In most cases the standard deviations of the data were statistically similar between vaccinated groups (Brown–Forsythe test) but they were different between the vaccinated and the non-vaccinated groups. The correlation analysis between the immune response data versus the individual clinical scores, the viral RNA AUC and the pfu AUC was done with the bilateral Spearman's non-parametric test. The PCA was done with 31 factors and included the individual viral RNA copies/ml at 2, 3, 4, 5 and 6 dpc, the viral RNA AUC, the individual pfu/ml at 2, 3 and 4 dpc, the pfu AUC, the individual clinical scores, the integrated temperature measurements, the T-cell responses at week 12, the anti-eGn IgG OD signals at 0, 5, 6, 8, 10 and 12 dpc, the anti-N IgG OD signals at 5, 6, 8, 10 and12 dpc, the anti-RVFV IgG OD signals at 0, 5, 6, 8, 10 and 12 dpc and the NAb titers at 10 dpc. PCA graphics has been produced using FactoMineR R package (http://factominer.free.fr/).

### Data availability

All datasets and tools developed by the authors are available to readers. Samples for RVFV-infected sheep and vaccinated sheep will require legal authorization from the Agence Nationale de la Sécurité du Médicament (ANSM), as RVFV is a “select agent” in this country. The ScFv sequences were deposited on Bankit (https://www.ncbi.nlm.nih.gov) under the ID 2070859 (release on 1 September 2018).

## Electronic supplementary material

Supplemental material
