# Supplementary material for: A Rift Valley fever virus Gn ectodomain-based DNA vaccine induces a partial protection not improved by APC targeting
Source: NPJ Vaccines. 2018 Apr 20;3:14. doi: 10.1038/s41541-018-0052-x (PMC5910381; doi:10.1038/s41541-018-0052-x)
Supplement: Supplementary file 1 — Supplemental material [file 41541_2018_52_MOESM1_ESM.pdf]

Supplementary material 1

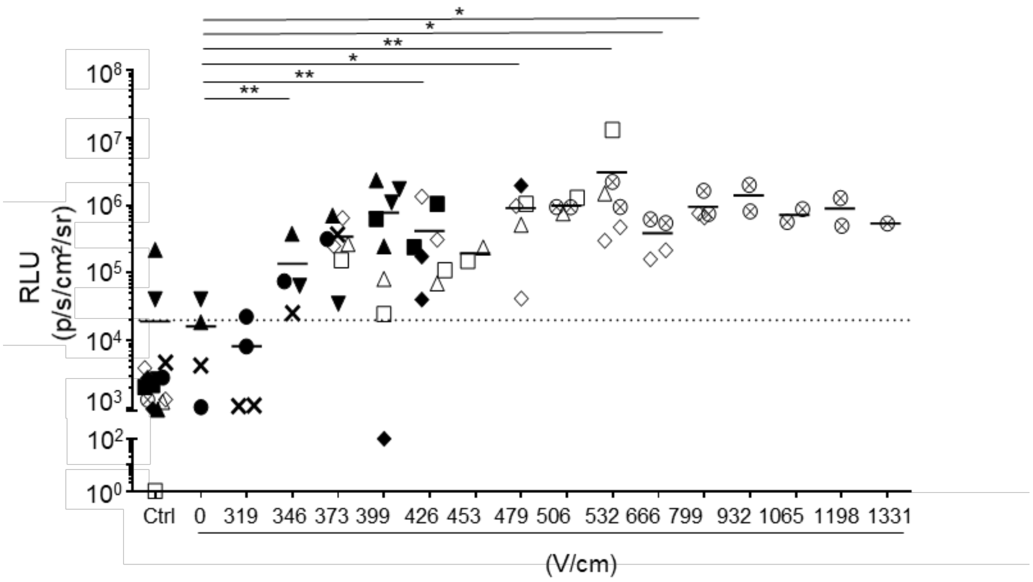

Optimization of the SEP V/cm parameter for pLuc expression in sheep skin *in vivo.* pLuc (100 µg) was injected intradermally in sheep skin followed by SEP (6 pulses, 10 ms per pulse with 90 ms intervals). Different voltages per cm pulses were applied. After 48 h, the site of SEP was harvested and processed to measure the luciferase activity (p/s/cm²/sr). The background signal is given by the non-treated skin (ctrl). The luciferase activity of the skin biopsies injected with pLuc and subjected to different V/cm from a given sheep is represented by the same symbol. The luciferase activity mean obtained at a given V/cm from different sheep is shown as a horizontal bar. P values were determined according to the Mann-Whitney test (*, $p < 0.05$; **, $p < 0.01$).

Supplementary material 2
Scoring index of the clinical signs

Clinical signs	Score
Anorexia	0-3
Eye aspect	0-3
Activity/movement	0-3
Lack of rumination	0-3
Nasal mucus	0-3
Depression/prostration	0-3
Respiratory movement	0-3
Tremor	0-3
Death	15

Supplementary material 3

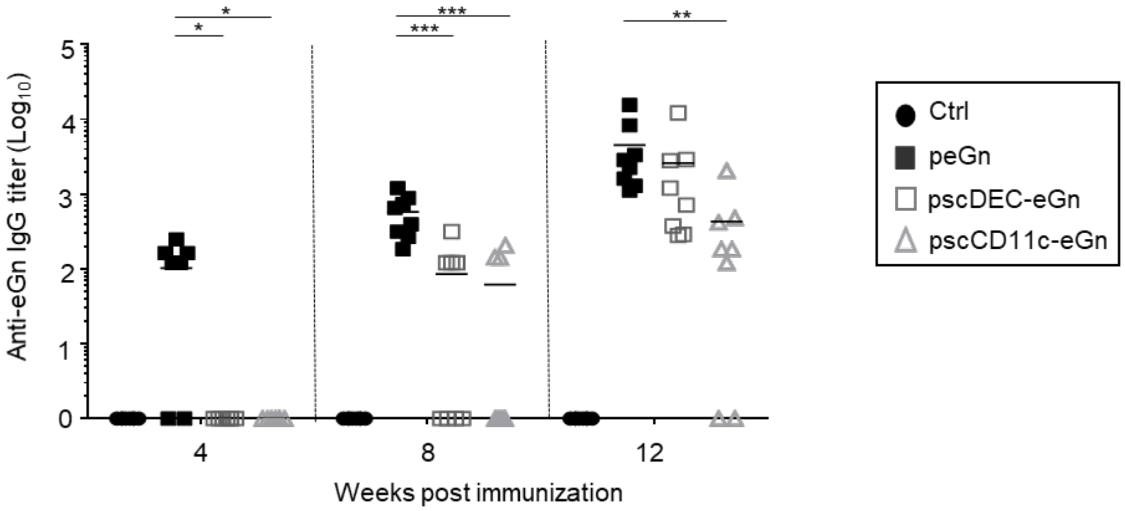

Anti-eGn IgG titers in sera of lambs vaccinated with peGn, pscDEC-eGn and pscCD11c-eGn. The serum of the lambs immunized with peGn, pscDEC-eGn, pscCD11c-eGn and of control lambs were assayed for the detection of eGn-specific IgG using indirect ELISA as described Fig. 3. Sera (1:100) were serially diluted 1:1. Endpoint titers were calculated with a regression analysis plotting dilution versus A_{450} as the highest dilution giving twice the absorbance of the negative control. Each symbol represents an individual animal and the mean is indicated. P values between 2 groups were determined according to the Mann-Whitney test (*, $p < 0.05$; **, $p < 0.01$; ***, $p < 0.001$).

Supplementary material 4

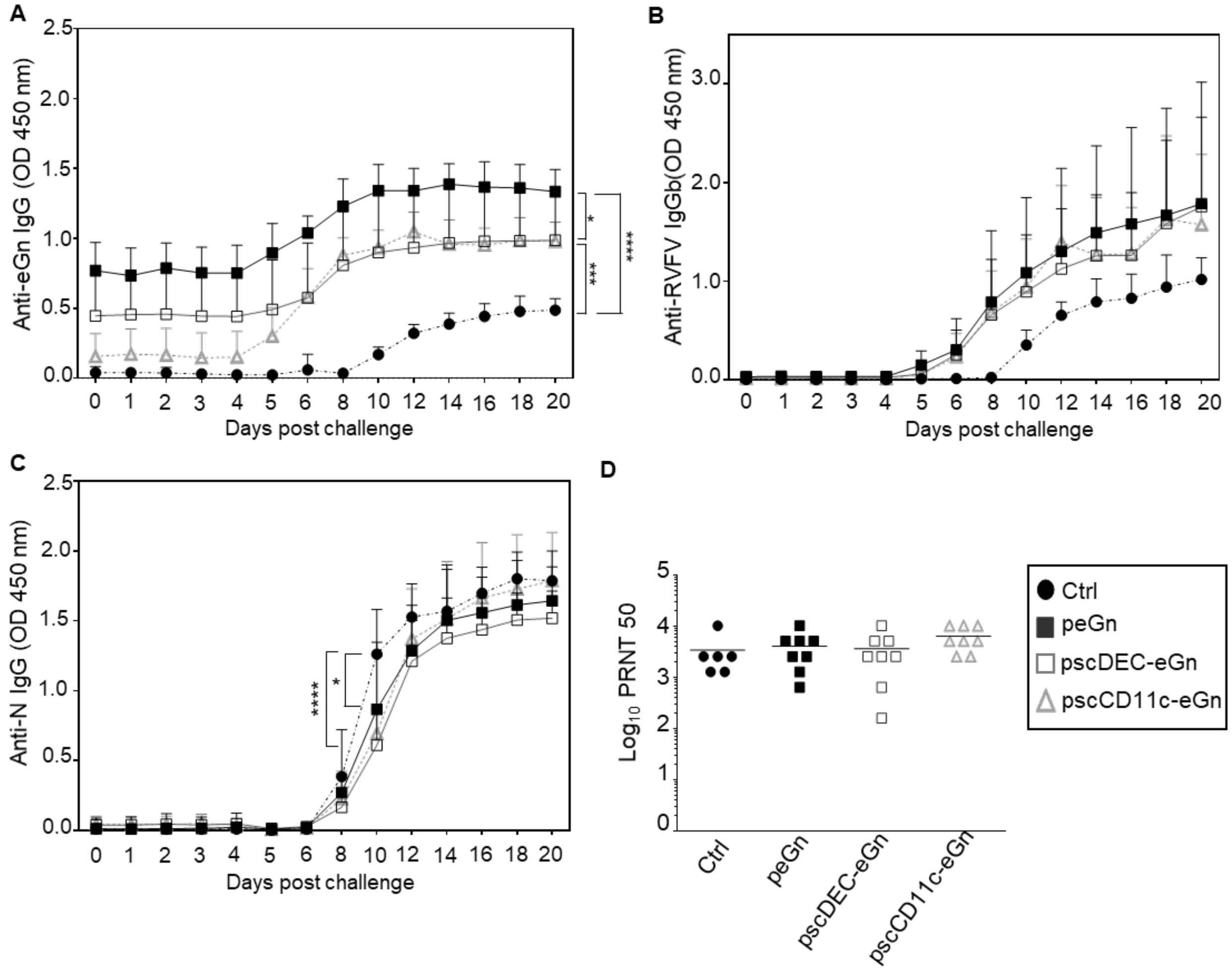

Anti-RVFV specific IgG responses after viral challenge in control lambs and lambs immunized with peGn, pscDEC-eGn and pscCD11c-eGn. Serum of peGn, pscDEC-eGn and pscCD11c-eGn immunized lambs and of control lambs were collected at 0, 1, 2, 3, 4, 5, 6, 8, 10, 12, 14, 16, 18, 20 dpc. Anti-eGn **(A)**, anti-RVFV **(B)** and anti-N **(C)** were measured by ELISA. Each distinct symbol represents the mean ± SD of the OD values obtained from individual sheep sera (1:100 dilution), n = 8 sheep per group. P values were determined according to the two-way Anova test with Bonferroni's correction to evaluate the statistical significance of the OD value differences between the vaccinated groups at different time points. The p values at 10 dpc for the anti-eGn, anti-RVFV and anti-N responses are indicated for clarity (*, $p < 0.05$; **, $p < 0.01$; ****, $p < 0.0001$). Neutralizing antibodies at 10 dpc **(D)** were detected with a plaque assay and titers were established as the last dilution which inhibited 50% of the foci number per well compared to virus-only control titration. P values were determined according to the Mann-Whitney test and no significant differences were measured between groups.

Supplementary material 5
Correlation analysis[1] between the immune response and the clinical and virological data

	dpc	Individual clinical score	Viral RNA AUC	pfu AUC
Anti-eGn	0	-0.49*[1]	-0.55**	-0.60**
	5	-0.37	-0.46*	-0.50*
	6	-0.31	-0.36	-0.36
	8	-0.34	-0.28	-0.28
	10	-0.33	-0.20	-0.23
	12	-0.28	-0.24	-0.30
Anti-RVFV	0	-0.50*	-0.44*	-0.52**
	5	-0.32	-0.37	-0.48*
	6	0.05	-0.15	-0.12
	8	0.24	0.26	0.22
	10	0.24	0.36	0.30
	12	0.28	0.36	0.23
Anti-N	5	-0.31	-0.17	-0.18
	6	-0.11	-0.02	-0.08
	8	-0.16	0.02	0.03
	10	-0.19	0.24	0.27
	12	0.20	0.62**	0.55**
T cell response	-	0.00	-0.12	-0.13
Nabs titers	10	0,15	0,28	0,27

[1]bilateral Spearman correlation analysis (*, $p < 0.05$; **, $p < 0.01$).
The r coefficient is reported.
